# Ligand-Based Design of Novel Quinoline Derivatives as Potential Anticancer Agents: An In-Silico Virtual Screening Approach

**DOI:** 10.3390/molecules29020426

**Published:** 2024-01-15

**Authors:** Khaoula Mkhayar, Ossama Daoui, Rachid Haloui, Kaouakeb Elkhattabi, Abdelmoula Elabbouchi, Samir Chtita, Abdelouahid Samadi, Souad Elkhattabi

**Affiliations:** 1 Laboratory of Engineering, Systems and Applications, National School of Applied Sciences, Sidi Mohamed Ben Abdellah-Fez University, Fez 30000, Morocco; khaoula.mkhayar@usmba.ac.ma (K.M.); ossama.daoui@usmba.ac.ma (O.D.); rachid.haloui@usmba.ac.ma (R.H.); 2 Laboratory for Oral Biology and Biotechnology Research, Department of Fundamental Sciences, Faculty of Medicine Dentistry, Mohammed V University, Rabat 10106, Morocco; kaouakeb.elkhattabi@fmd.um5.ac.ma; 3 Euromed Research Center, Euromed Faculty of Pharmacy, Euromed University of Fes (UEMF), Meknes Road, Fez 30000, Morocco; a.elabbouchi@ueuromed.org; 4 Laboratory of Analytical and Molecular Chemistry, Faculty of Sciences Ben M’Sik, Hassan II University of Casablanca, Casablanca P.O. Box 7955, Morocco; samirchtita@gmail.com; 5 Department of Chemistry, College of Science, United Arab Emirates University, Al Ain P.O. Box 15551, United Arab Emirates

**Keywords:** quinoline, anti-gastric cancer, 3D-QSAR, CoMFA, POM, ADME-Tox, molecular docking, molecular dynamics, MM-GBSA

## Abstract

In this study, using the Comparative Molecular Field Analysis (CoMFA) approach, the structure-activity relationship of 33 small quinoline-based compounds with biological anti-gastric cancer activity in vitro was analyzed in 3D space. Once the 3D geometric and energy structure of the target chemical library has been optimized and their steric and electrostatic molecular field descriptions computed, the ideal 3D-QSAR model is generated and matched using the Partial Least Squares regression (PLS) algorithm. The accuracy, statistical precision, and predictive power of the developed 3D-QSAR model were confirmed by a range of internal and external validations, which were interpreted by robust correlation coefficients ($R_{Train}^{2}=0.931$; $Q_{cv}^{2}=0.625$; $R_{Test}^{2}=0.875$). After carefully analyzing the contour maps produced by the trained 3D-QSAR model, it was discovered that certain structural characteristics are beneficial for enhancing the anti-gastric cancer properties of Quinoline derivatives. Based on this information, a total of five new quinoline compounds were developed, with their biological activity improved and their drug-like bioavailability measured using POM calculations. To further explore the potential of these compounds, molecular docking and molecular dynamics simulations were performed in an aqueous environment for 100 nanoseconds, specifically targeting serine/threonine protein kinase. Overall, the new findings of this study can serve as a starting point for further experiments with a view to the identification and design of a potential next-generation drug for target therapy against cancer.

## 1. Introduction

Cancer remains a leading cause of global mortality, as reported by the World Health Organization [1,2]. The pharmaceutical industry continues to dedicate extensive research efforts to anti-cancer drugs [3]. Recent advancements in medicinal and therapeutic compounds have been significantly influenced by heterocyclic compounds, owing to their diverse substituent options, low toxicity, and high efficacy. Among these, quinoline compounds have garnered significant attention for their potential as anticancer agents, as they are widely distributed in nature and can be extracted from specific plants [4]. The therapeutic applications of quinoline nuclei are extensive, and quinolone derivatives exhibit a wide range of pharmacological effects [5]. Several novel therapeutic agents have been developed based on the quinoline nucleus, highlighting the potential of quinoline and its derivatives in drug discovery. The broad spectrum of biological and pharmacological activities associated with quinoline and its derivatives has led to the exploration of numerous synthetic routes. serine/threonine kinase STK10, also known as Aurora-C kinase, is a protein kinase that plays a crucial role in several cellular processes [6], particularly in cell division and the regulation of the cell cycle. Its importance lies in its ability to phosphorylate and modulate various substrates, which in turn influences critical cellular functions [7]. Its implications for cancer and other diseases make it a subject of significant research and drug development efforts [8,9].

Pharmaceutical industries are employing novel techniques, such as quantitative structure-activity relationships (QSAR), to predict the activity of compounds before synthesis. The use of 3D quantitative structure-activity relationships (3D-QSAR) for the design and development of potent drugs has proven highly advantageous [10]. This method establishes a correlation between the 3D structural features of a chemical and its property of interest. The ligand-based approach has been a well-established method since the late 1980s [11] and is a well-established method for developing a predictive model. A ligand-based drug design can be improved by evaluating the structural and electrostatic properties of the ligands corresponding to their activity using comparative molecular field analysis (CoMFA). This study establishes 3D-QSAR models based on 33 compounds (Quinoline Derivatives analogues as gastric cancer cell lines) [12] and correlates their three-dimensional structures with their biological activity through comparative molecular field analysis (CoMFA). The results obtained from the combined computational approach have facilitated the development of novel anticancer agents. Additionally, we conducted other steps, including Petra-Osiris-Molinspiration (POM) studies, to identify and determine the pharmacophore site that influences the biological activity upon chemical substitution. Molecular docking, a commonly employed method in drug design, was utilized to predict the interaction between small molecules and the target binding sites. In this study, docking was employed to investigate the interaction between the target protein (6I2Y) and the newly designed ligands, as well as the reference ligand (with a high pIC_50_), to validate the results obtained from CoMFA. Furthermore, we performed molecular dynamics simulations of the most active synthesized molecule and the optimally designed molecule to gain further insights.

## 2. Results and Discussions

### 2.1. Molecular Alignment

**Figure 1:**
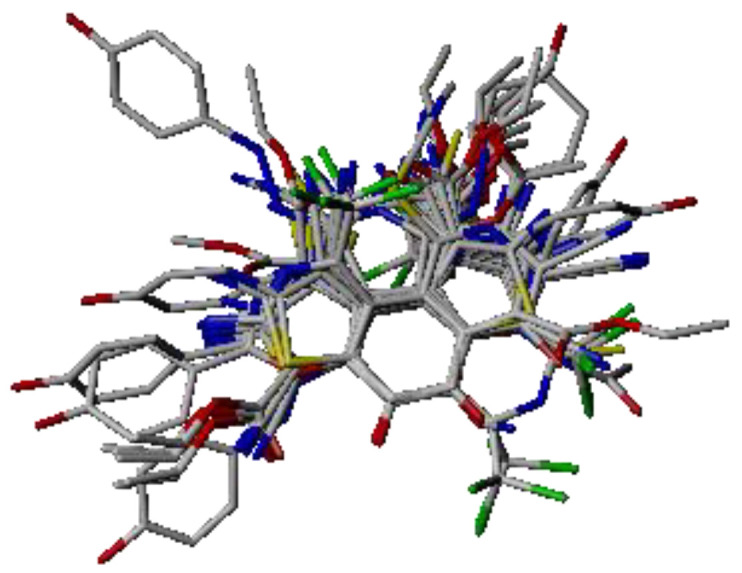
Alignment of dataset using ligand **22c** as a template.

Figure 1 depicts the alignment of the 33 ligands, specifically quinoline derivatives, with ligand 22c serving as the reference compound.

### 2.2. Comparative Molecular Field Analysis

Comparative Molecular Field Analysis (CoMFA) was conducted on a training set consisting of 24 ligands from the total of 33 quinoline derivatives, which were reported to exhibit Serine/threonine kinase STK10 inhibition based on 3D-QSAR modeling. The CoMFA results revealed satisfactory coefficients of cross-validated correlation (Q^2^) with a value of 0.625, indicating good predictive ability. Additionally, high coefficients of determination (R^2^) were obtained, with a value of 0.913, indicating a strong correlation between the predicted and observed values. The low standard error of estimate (SEE) values, coupled with the optimal selection of three components, further validate the model’s reliability and predictive power. The external predictive ability of the model was also assessed using a test set of nine randomly selected molecules, yielding a r_ext_^2^ value of 0.875 for CoMFA.

**Figure 2:**
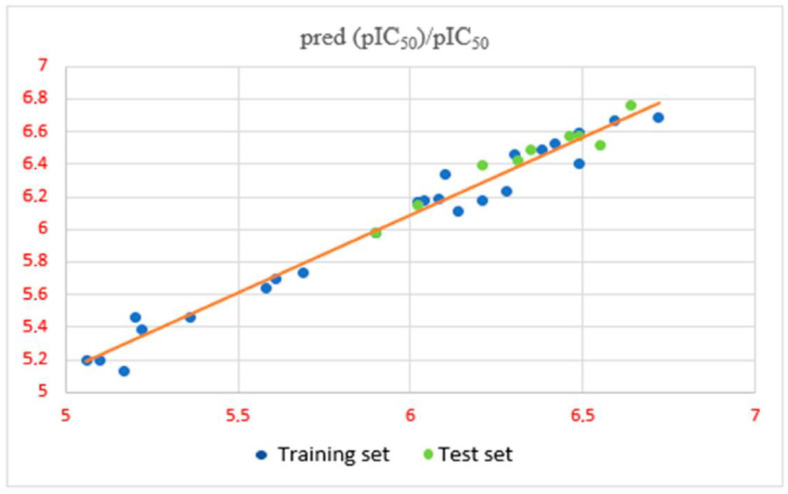
pIC_50_ obs/pIC_50_ pred by the CoMFA model.

The CoMFA model suggests that both steric and electrostatic fields significantly contribute to the binding affinity, with steric and electrostatic contributions accounting for 51.5% and 48.5%, respectively. The quantitative results presented in Table 1 demonstrate that the CoMFA model provides reasonable and reliable predictions for inhibitor activity. Furthermore, Figure 2 illustrates a significant correlation between the calculated and observed pIC_50_ values.

The values of the predicted biological activities of the compounds in the series are represented in Table 2.

### 2.3. CoMFA Contour Map Analysis

**Figure 3:**
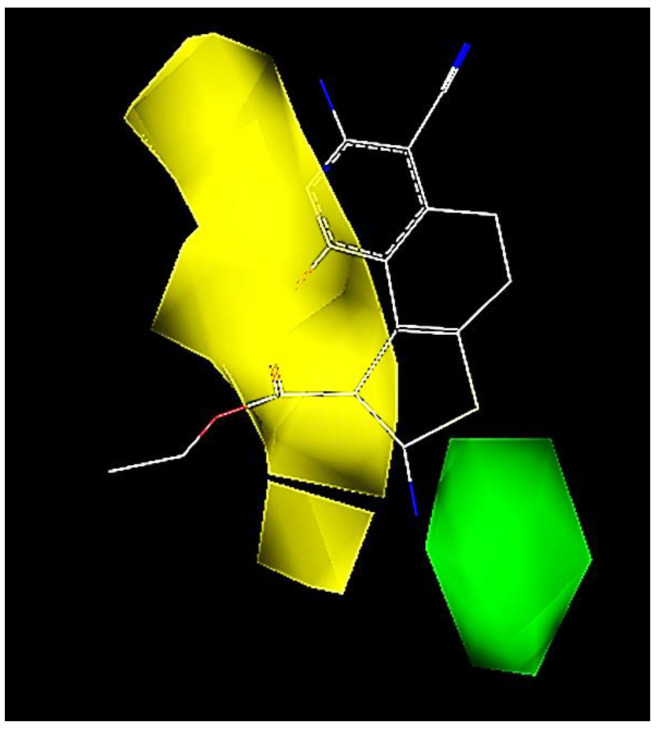
Std* coeff. contour maps of CoMFA analysis with 2 Å grid spacing in combination with ligand 22c.

Maps of CoMFA contours were generated to provide insights into the regions surrounding the ligands where changes in each field were predicted to impact the activity. These maps visually illustrate the areas where modifications in the steric and electrostatic fields are likely to result in increased or decreased activity. These contour maps help in understanding the structural requirements for optimal binding and can guide the design of new ligands with enhanced activity. In CoMFA, the steric and electrostatic field descriptors contribute approximately 51.5% and 48.5%, respectively. This suggests that both descriptors significantly impact the increase or decrease of cancer inhibitory activity. The steric contour map generated by CoMFA is depicted in Figure 3. The green contours (contributing 80%) indicate the regions where bulky clusters have a positive impact on cancer inhibitory activity, while the yellow contours (contributing 20%) suggest the areas where bulky groups decrease inhibitory activity.

**Figure 4:**
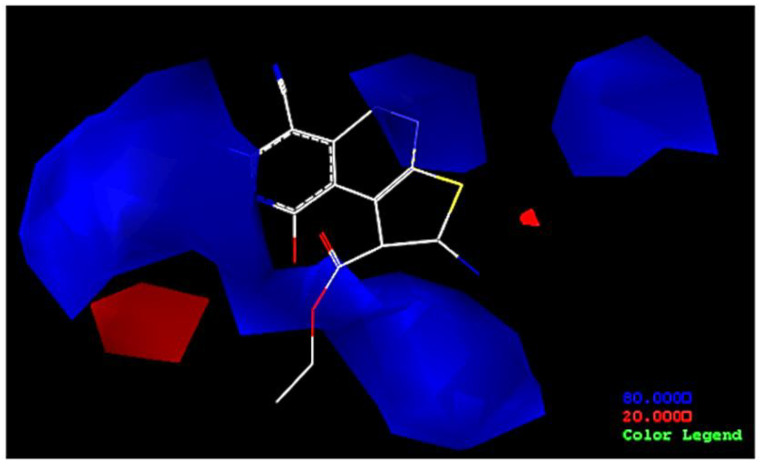
Std* coeff. Contour maps of CoMFA analysis with 2 Å grid spacing in combination with ligand 22c.

The green contour near the R_1_ position indicates that the presence of bulky groups at this site is favorable and may potentially increase the activity of the ligands. On the other hand, the yellow contour near R_2_ suggests that bulky groups at this position could decrease the potency. These observations explain why ligands like 22c, with R_1_ = COOEt and R_2_ = CN, exhibit higher activity, while ligands such as 22b, with R_1_ = CN and R_2_ = COOEt, and 22d, with R_1_ = COOEt and R_2_ = COOEt, show lower activities with −LogIC50 values of 5.9 and −6.46, respectively. The contour maps provide valuable insights into the structural requirements for optimal binding and help explain the varying activities of the ligands. Figure 4 illustrates the CoMFA electrostatic contour map, where blue contours (with an 80% contribution) indicate regions where the presence of electronegative groups positively impacts cancer inhibitory activity. In contrast, red contours (contributing 20%) delineate areas where the presence of electronegative groups negatively affects cancer inhibitory activity. The presence of a red contour near the R_1_ position suggests that groups with positive charges may enhance the activity of the ligands. Similarly, a large blue contour near the R^2^ position indicates that groups with negative charges in these zones increase the activity. This explains why ligands like 24c with R_1_ = CN and R_2_ = SCN and 24f with R_1_ = COOEt and R_2_ = SCN exhibit high activity. The interaction of positive and negative charges at these specific positions contributes to the favorable binding and increased potency of these ligands.

### 2.4. Y-Randomization Test

In order to validate the CoMFA model, Y-Randomization was used. The dependent variable was shuffled several times, and then a 3D-QSAR was developed after each shuffle. The results are presented in Table 3. The Q^2^ and R^2^ values were lower than those of the initial CoMFA model, indicating the robustness of the QSAR model, which was not obtained by random correlation.

### 2.5. ROC-AUC Analysis

**Figure 5:**
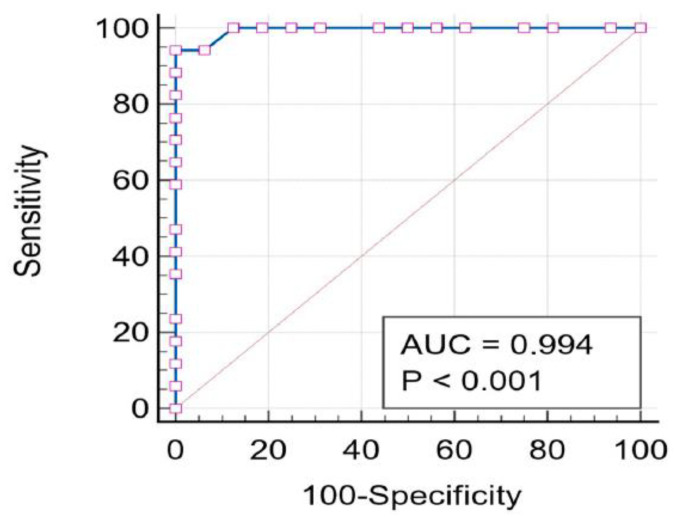
ROC curve.

Figure 5 presents the validation ROC curve, assessing the predictive accuracy of the QSAR model for the biological activities of molecules. Referring to this figure, we observe an ROC curve positioned near the upper-left corner, indicating a high predictive capacity. It reveals an outstanding AUC value of 0.994, demonstrating the model’s efficiency in accurately differentiating active from inactive compounds (*p* < 0.001).

### 2.6. Design of New Quinoline-Based Ligands

According to the significant information obtained from the study of contour maps (the 3D-QSAR/CoMFA model), we determined the regions of modification to enhance the inhibitory activity. From this study, we proposed and designed seven derivative anticancer agents by modifying the chemical structure of molecule 22c (the most active molecule in the database), as shown in Table 4. Ligand 22c was used as a template to optimize and align these newly designed ligands with the existing database. The aim was to create ligands that exhibit improved activity and binding affinity, taking inspiration from the structural features and properties of ligand 22c.

**Scheme 1:**
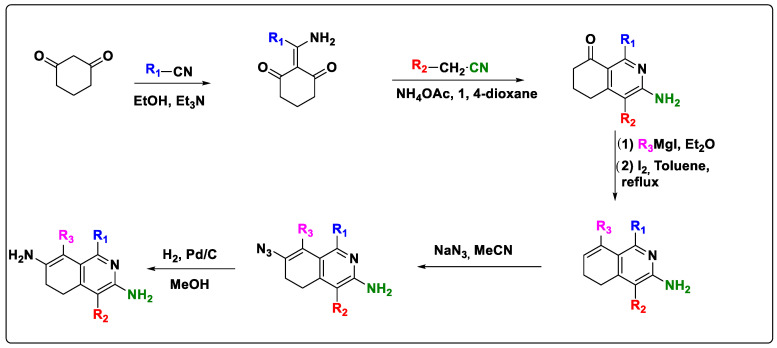
Synthetic pathways adopted in the design of the proposed new molecular structures.

These seven new ligands have been designed by suggesting different reaction mechanisms based on the literature [13]. The cyclohexan-1,3-dione was chosen as the model substrate for the synthesis of the proposed new molecular structures, as described in the synthetic pathways in Scheme 1. The first step involves a condensation reaction in the presence of nitrile derivatives and triethylamine in an ethanol solution. The second step is intramolecular cyclization due to the presence of acetonitrile derivatives and ammonium acetate in 1,4-dioxane. Subsequently, the Grignard reaction breaks the cyclic ketone, breaking C-O and forming C-R_3_, followed by iodination and elimination reactions. The next step involves an azide reaction using sodium azide as the source of azide. In the final step, a reduction reaction occurs in the presence of Pd/C in a methanol solution.

### 2.7. POM Results

The POM program was used to calculate molecular properties essential for QSAR analysis, bioactivity prediction, and toxicity prediction. Table 5 presents the results of Osiris calculations, which include the assessment of various toxicities (mutagenicity, tumorigenicity, irritation, reproduction) and physicochemical properties (cLogP, solubility, drug-likeness, and Drug-score) for the ligands. The Osiris methodology provides a framework for evaluating toxicities and identifying potential risks associated with specific molecular fragments. Toxicity alerts indicate whether the drawn structure may pose a risk based on predefined risk categories.

From Table 5, it is evident that the designed ligands exhibit non-mutagenic, non-tumorigenic, non-irritant, and no reproductive effects. Furthermore, the cLogP values of the ligands are within a reasonable range, as they do not exceed 5.0, indicating a favorable probability for good absorption [14]. All designed ligands possessed cLogP values in the acceptable range. So these ligands are expected to have good bioavailability. The aqueous solubility of a ligand is a crucial factor in its absorption and distribution within the body. Poor solubility is often correlated with inadequate absorption, so it is generally advisable to avoid ligands with low solubility. Our estimated solubility value (S) represents the logarithm (base 10) of a ligand’s solubility in moles per liter (mol/L). It is worth noting that over 80% of drugs available on the market have a solubility value greater than −4. According to our findings, the ligands 1, 3, 5, and 8 exhibit low solubility with values below −5.

The designed ligands generally demonstrate low to moderate drug scores (DS < 0.50), except for ligand 7 with a drug-likeness value (DL) of −4.15. Based on our stringent criteria, which consider GPCR ligands, ion channel modulators, kinase inhibitors, and nuclear receptor ligands, these ligands are anticipated to exhibit activity similar to that of the standard drugs employed. Table 6 presents the calculation of cLogP (octanol/water partition coefficient) using the robust methodology developed by Molinspiration. This method is highly reliable and capable of processing a wide range of organic and organometallic molecules. Additionally, the Total Polar Surface Area (TPSA) is determined using the methodology published by Ertl et al. [15]. Polar fragments containing nitrogen (N) and oxygen (O) atoms are taken into account. The designed ligands exhibit numerous NH—O or N—HO interactions, which contribute to their high bioavailability. The drug-likeness of the designed ligands appears to be appropriate and falls within the same range as standard drugs, as indicated by their negative values.

### 2.8. Molecular Docking Results

**Figure 6:**
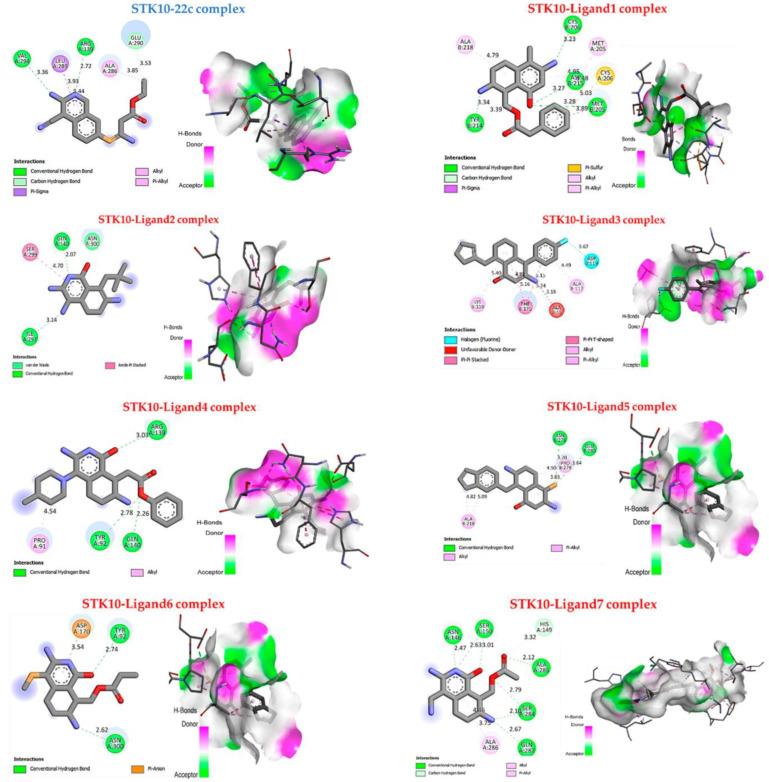
2D and 3D interactions of the optimized ligands 1–7 and template 22c with STK10 receptor (PDB ID code: 6I2Y).

The Auto-Dock software (ADT) MGLTools 1.5.6 packages were used to explore the interaction of the active ligand synthesized (22c) and the designed ligands (1 to 7) with the Serine/threonine kinase STK10 (PDB code: 6I2Y). We used Discovery Studio 2021 software (v21.1.0.20290) to visualize the types of interactions that were created between the most active ligand 22c, the designed ligands, and the receptor 6I2Y (Table 7). Figure 6 shows the 2D and 3D interactions of ligands 1, 2, 3, 4, 5, 6, 7, and 22c with STK10 (PDB ID code: 6I2Y). The binding affinity values of (1 to 7) are −6.8 kcal/mol, −5.1 kcal/mol, −7.5 kcal/mol, −6.3 kcal/mol, −5.6 kcal/mol, −5.2 kcal/mol, and −7.9 kcal/mol, respectively.

The reference ligand 22c has a binding affinity value of −4.3 kcal/mol. This indicates that the designed ligands are more stable in the protein pocket than the reference ligand. The reference 22c has two conventional hydrogen bonds: a hydrogen bond Val A294 with the nitrogen attached to the benzene ring at a distance of 3.63 Å, a hydrogen bond Arg A139 with the nitrogen attached to the benzene ring at a distance of 2.72 Å, and a π-sigma bond Leu A289 with the benzene ring at a distance of 5.44 Å. One carbon hydrogen bonds GluA290 with a different benzene ring at a distance of 5.31 Å, and alkyl and π-alkyl bonds Ala A286.

The designed ligand 1 forms critical interactions with the protein through seven active sites. These interactions involve several residues, namely Cys A206, Asp B215, Met B205, and Tyr B214. Hydrogen bonds are formed between these residues and the ligand’s nitrogen and hydroxyl groups, with distances ranging from 3.23 Å to 4.95 Å. Additionally, a π sulfur bond is observed between the Cys B206 residue and the ligand, spanning a distance of 5.03 Å. Moreover, the residues Ala B286 and Met B205 form π-alkyl and alkyl interactions, respectively, with the ligand’s alkene and benzene ring, with distances of 4.79 Å and 4.95 Å.

The designed ligand 2 forms crucial interactions with the protein through four active sites. Gln A140 and Ile A297 residues establish hydrogen bonds with the nitrogen atom, enhancing the stability of the complex. Additionally, the Ser A299 residue contributes to the interaction through amide stacking, further reinforcing the binding. Furthermore, the Asn A300 residue engages in van der Waals interactions with the hydroxyl group, adding to the overall stability and specificity of the binding. These various types of interactions are of great importance as they play key roles in facilitating the molecular recognition and binding between the designed ligand and the protein.

The designed ligand 3 and the protein are connected through four active sites, each contributing to the binding interaction. The Phe176 residue forms a π-stacked interaction with the designed ligand at a distance of 5.03 Å, providing stability to the complex. Additionally, the Ala B117 and Lys B159 residues engage in π-alkyl and alkyl interactions, respectively, with the benzene ring of the ligand at distances of 4.49 Å and 5.40 Å. These interactions contribute to the overall binding strength. Furthermore, the Ser B299 residue forms a halogen bond with the ligand at a distance of 3.63 Å, further enhancing the stability of the complex. Lastly, the Phe B176 residue forms a π-π stacked bond with the ring of the ligand, adding to the specific molecular recognition.

The designed ligand 4 forms crucial interactions with the protein through four active sites. Firstly, there are hydrogen bonds formed between Arg A139, Tyr A92, and Gln A140 residues with the oxygen attached to the benzene ring of the ligand. These hydrogen bonds contribute to the stability and specificity of the binding. Additionally, an alkyl bond and a π-alkyl bond are observed with the Pro A91 residue. These interactions, involving hydrophobic and aromatic interactions, respectively, further enhance the binding between the designed ligand and the protein. Collectively, these various types of interactions play a significant role in facilitating the molecular recognition and binding between the designed ligand 4 and the protein.

The designed ligand 5 establishes important interactions with the protein through four active sites. Two conventional hydrogen bonds are formed, with Glu B280 and Asn B278 residues interacting with the sulfur atom attached to the benzene ring at distances of 3.73 Å and 3.64 Å, respectively. These hydrogen bonds contribute to the stability and specificity of the binding between the ligand and the protein. Additionally, an alkyl bond and a π-alkyl bond are observed with the Ala B218 and Pro B279 residues, respectively. These interactions, involving hydrophobic and aromatic interactions, further enhance the binding between the designed ligand 5 and the protein.

The designed ligand 6 establishes vital interactions with the protein through three active sites. Notably, two conventional hydrogen bonds are formed between the TYR A92 and Asn A300 residues and the sulfur atom attached to the benzene ring of the ligand. These hydrogen bonds occur at distances of 3.54 Å and 2.74 Å, respectively, underscoring their significance in stabilizing the ligand-protein interaction. In addition, there is a π-anion bond observed with the ASN A300 residues.

The designed ligand 7 forms crucial interactions with the receptor through six conventional hydrogen bonds. These hydrogen bonds involve Asn A146, Ser A150, Ala A285, Ser A284, and Gln A287 Val A294 residues, interacting with the nitrogen attached to the benzene ring of the ligand. The distances between the donor and acceptor atoms in these hydrogen bonds are 2.47 Å, 2.63 Å, 2.12 Å, 2.10 Å, and 2.67 Å, respectively. Additionally, there is a hydrogen bond formed between Arg A139 and the nitrogen attached to the benzene ring at a distance of 2.72 Å. Furthermore, a carbon-hydrogen bond is observed between His A149 and a different benzene ring of the ligand, with a distance of 3.32 Å. Additionally, there are alkyl and π-alkyl bonds formed with the Ala A286 residue, with a distance of 3.75 Å.

These various types of interactions, including hydrogen bonding, carbon-hydrogen bonding, and hydrophobic interactions, contribute to the stability and specificity of the binding between the designed ligand 7 and the receptor. Collectively, these interactions play a significant role in facilitating molecular recognition and binding between the ligand and the receptor. These interactions are the main factors having an important impact on the affinity of a ligand to a receptor, which is in line with results from hydrophobic and electrostatic contour maps, indicating that the proposed ligand 7 could exert a strong inhibitory effect on Serine/threonine kinase STK10 in its chosen pose.

### 2.9. Molecular Dynamics Simulations

**Figure 7:**
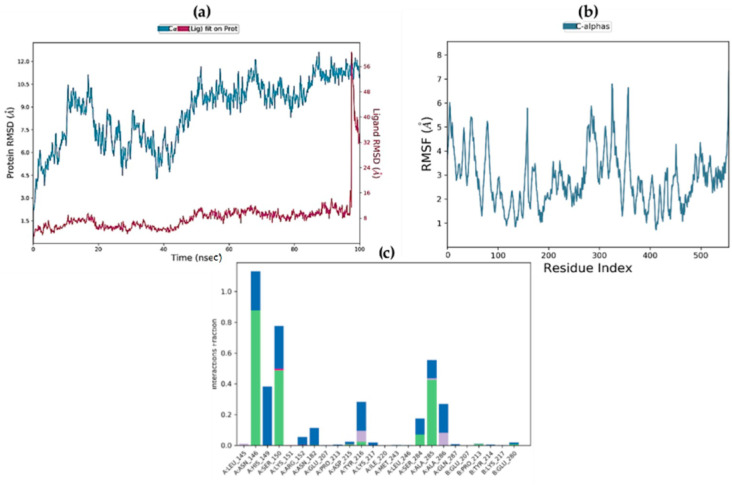
Molecular dynamics simulation for the complex STK10-Ligand7 in terms of (**a**) RMSD timeline motion; (**b**) RMSF timeline motion; and (**c**) protein-ligand timeline contacts.

**Figure 8:**
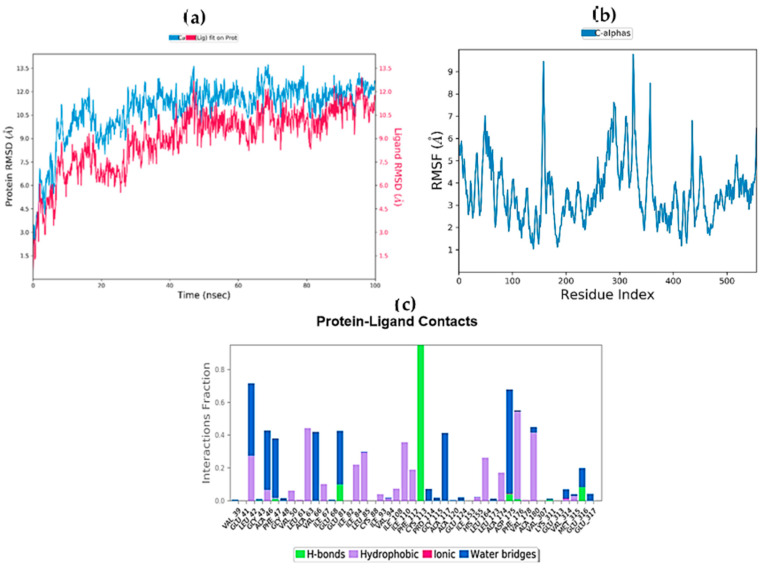
Molecular dynamics simulation for the complex STK10-co-crystallized ligand in terms of (**a**) RMSD timeline motion; (**b**) RMSF timeline motion; and (**c**) protein-ligand timeline contacts.

Molecular dynamics (MD) simulations were conducted to validate the results obtained from 3D-QSAR and molecular docking. MD serves as a powerful tool to assess the dynamic stability of a system [16]. In our study, we conducted a 100 ns MD simulation on the optimal docking configurations (complex 7) and the co-crystallized ligand. Two parameters, namely root mean square deviation (RMSD) and root mean square fluctuation (RMSF), were utilized to evaluate the structural fluctuations in protein 6I2Y (Figure 7 and Figure 8). RMSD helps assess the similarity or deviation between different molecular structures over time. Figure 7a and Figure 8a illustrate the RMSD of protein 6I2Y (depicted in blue) and ligand 7 as well as co-crystallized ligand (depicted in red). Throughout the simulation, no significant change was observed in the RMSD for ligand 7. However, a remarkable change was noted for the co-crystallized ligand over time. This indicates that ligand 7 is more stable than the co-crystallized ligand.

RMSF, on the other hand, is a useful metric for characterizing local changes along the protein chain [13]. RMSF (Root Mean Square Fluctuation) is a measure of the deviation of the position of each atom in a protein from its average position over a simulation. The RMSF values of the STK10-Ligand7 complex ranged from 1 to 6.5 Å (Figure 7b), while the RMSF values of the STK10-co-crystallized complex ranged from 1 to 9.5 Å (Figure 8b). The RMSF values of the designed ligand are lower than those of the co-crystallized compound, indicating that it is more stable. Figure 7c and Figure 8c display the protein-ligand interactions, including hydrogen bonds, hydrophobic contacts, and water bridges. Notably, hydrogen bonds (H-bonds) play a significant role in ligand binding. involving amino acids such as Asn146, Ser150, Tyr216, Ser284, Ala285, and Pro213. Hydrophobic interactions were observed with Tyr216 and the Leu145-Ala286 residues. During the simulation trajectory, specific amino acids came into contact with the co-crystallized ligand, including Glu 68, Cys 113, Asp 145, Leu 42, Ala 46, Val 50, Ile 61, and Ile 180.

These molecular dynamics simulations offer valuable insights into the dynamic behavior of the complex STK10-Ligand7 and the complex STK10-co-crystallized ligand, confirming the stability of ligand 7.

### 2.10. MM/GBSA Free Energy Calculation

Table 8 presents the obtained results of the energies associated with the interaction between the STK10 protein, ligand7, and compound 22c, respectively. The value of ΔG_bind_ (−20.9293 kcal/mol and −13.6075 kcal/mol) indicates the overall binding free energy. This negative value implies a favorable binding interaction between the STK10 protein and ligand7. The value of ΔG_bind_ Hbond (−2.9044 kcal/mol and −1.1743 kcal/mol) specifies the contribution of hydrogen bonding to the binding free energy. It suggests that hydrogen bonding significantly contributes to the stability of the protein-ligand interaction. And the value of ΔG_bind_ VdW (−0.3727 kcal/mol and 0.48926 kcal/mol) represents the van der Waals contribution to the binding free energy. This indicates the role of van der Waals forces in stabilizing the STK10-ligand7 complex, although to a lesser extent compared to hydrogen bonding. These results indicate a favorable binding affinity between STK10 and ligand 7, primarily driven by hydrogen bonding interactions, followed by van der Waals forces, demonstrating the stability of ligand 7.

## 3. Materials and Methods

### 3.1. Focused Chemical Library

The dataset comprised 33 analogues of quinoline derivatives synthesized as anti-cancer agents by Mohareb et al. [17]. The dataset was divided into two sets, with 24 compounds selected as the training set and 9 compounds selected as the test set to assess the effectiveness of the obtained model. Table 9 displays the structures and biological activities of all the compounds in the training and test sets. A 3D-QSAR model was constructed, and their physicochemical properties were analyzed using this dataset.

### 3.2. Molecular Modelling

A molecular structure was drawn in SYBYL-X 2.0 software using the sketch module and then subjected to minimization using the Tripos force field [18]. Gasteiger-Hückel charges were assigned to the ligands, and the conjugate gradient method was employed with a gradient convergence criterion of 0.01 kcal/mol/Å. Molecular alignment is considered a crucial parameter in 3D-QSAR analysis because it is highly sensitive to the results. We conducted molecular alignment for the database, where molecules are superimposed on the common core using compound number 22c (the most active compound) as the model.

### 3.3. 3D-QSAR Modeling

One of the computational methods utilized in drug design is the three-dimensional quantitative structure-activity relationship (3D-QSAR) [19]. To construct predictive 3D-QSAR models based on molecular alignment, CoMFA studies were performed to investigate and analyze the electrostatic and steric contributions of the dataset [20]. Previous literature has described the use of 3D-QSAR in various studies [21]. In CoMFA, steric and electrostatic properties are computed based on Lennard-Jones and Coulomb potentials, respectively. The CoMFA analysis employed the Tripos force field, utilizing a spatial grid reference of 2 Å across all Cartesian axes. To compute steric and electrostatic energies, a sp^3^ hybridized carbon atom carrying a net charge of +1.0 was utilized. Default settings were applied for parameters such as the correction factor, set at 0.3 to regulate the Gaussian function slope, and the cutoff energy, defaulted to 30 kcal/mol [22].

### 3.4. PLS Analysis and Validations

To assess the linear correlation between the 3D-QSAR descriptors and the activity values of the biological variables, the partial least squares (PLS) regression method (Wold, 1991) was applied to the extensive variables obtained from the field calculations [22,23]. Cross-validation (Q^2^) was performed by excluding one compound from the training set and predicting its activity using the developed model with the remaining (N-1) compounds. This process was repeated for each compound until all were excluded once. Models with higher Q^2^ values, fewer components, and a low standard error of estimate (Scv) were chosen. Column filtering was set to 2.0 kcal/mol to reduce noise and expedite the analytical process [24]. Once the optimal number of components was determined, the final PLS model was deduced without validation [25] to calculate the maximum determination coefficient (R^2^).

### 3.5. Validation and Predictive Power of 3D-QSAR Model

Every QSAR study strives to develop a model that exhibits the highest level of predictive ability and generalizability. In order to evaluate the predictive capability of the 3D-QSAR models, nine compounds from a testing set were utilized. The 3D-QSAR model, constructed using the training set, was employed to predict the inhibitory activities of these ligands after aligning them using the same methods described earlier.

### 3.6. Y-Randomization Test

To assess the significance of the model, Y-randomization was employed to eliminate the potential presence of a chance association between specific descriptors and their corresponding activities [26]. The Y-randomization test ensures that the random correlation coefficient (Rr^2^) of randomly generated models is lower than the correlation coefficient of the original non-random model (R^2^) [27]. This procedure helps confirm the reliability and validity of the original model.

### 3.7. ROC-AUC Analysis

The ROC (Receiver Operating Characteristic) curve is a crucial tool to evaluate the performance of binary classification models [14]. It graphically represents sensitivity against specificity at various prediction thresholds. This visual display assesses a model’s ability to differentiate true positives from false positives. We typically generate the ROC curve using the MedCalc statistical software (http://www.medcalc.org (accessed on 27 December 2023)).

### 3.8. POM Analysis

Petra/Osiris/Molinspiration Analysis (POM) is a widely utilized approach for generating two-dimensional models that aid in identifying and characterizing pharmacophore sites responsible for changes in biological activity due to chemical substitutions. POM offers several advantages, including the ability to predict molecular biological activity and represent steric/electrostatic properties, as well as biological activities, using pharmacophore sites. This analysis provides valuable insights into ligand-receptor interactions and receptor topology [27]. In addition, we utilized Osiris, Petra, and Molinspiration to analyze the pharmacokinetic profiles of the proposed compounds. These tools were validated using a database of approximately 7000 drug molecules [28].

#### 3.8.1. Petra

The PETRA program package includes an empirical method for calculating the physicochemical properties of organic molecules [29]. These methods have been developed by Prof. J. Gasteiger’s research group over the course of 20 years. The program allows for the quantification of various chemical properties, including heats of formation, bond dissociation energies, inductive effect, π-charge distribution, sigma charge distribution, resonance effect, delocalization energies, and polarizability effect.

#### 3.8.2. Osiris

Structure-based design is widely used in drug discovery, but many potential drugs fail to progress to the clinic due to ADME-Tox (absorption, distribution, metabolism, excretion, and toxicity) liabilities. Among the enzyme classes implicated in ADMET issues, the cytochrome P450 enzyme family holds significant importance. Inhibition of these enzymes or the production of undesired metabolites can lead to adverse drug reactions. To address this, an online version of the crucial program “Osiris” is already available [30].

#### 3.8.3. Molinspiration

We utilized the (https://molinspiration.com/) [31] to obtain parameters such as MiLogP, TPSA, and drug likeness. The MiLogP parameter, calculated by the Molinspiration methodology, is determined by summing fragment-based contributions and correction factors. It serves as an indicator of good permeability across the cell membrane. The volume of a molecule is calculated by considering its contributing groups. TPSA, on the other hand, refers to the hydrogen bonding potential of compounds. The number of rotatable bonds in a molecule is a measure of its molecular flexibility and is indicative of drug absorption and bioavailability. Drug likeness data compares the structure and properties of a molecule with those of known drugs, providing insights into its molecular properties.

### 3.9. Molecular Docking

In recent years, molecular docking has emerged as a crucial tool in computer-assisted drug design for predicting binding affinity and analyzing the interaction mode of drugs. It offers improved efficiency and cost reduction in research. In this study, molecular docking was conducted using Discovery Studio 2016 software [32] and AutoDock software (ADT) MGLTools 1.5.6 packages [33]. We performed molecular docking on newly designed quinoline derivatives and the most active synthetic molecule to bind with the protein. Throughout this process, each compound produced nine distinct poses. These poses were meticulously exported to allow for a comprehensive examination of their various binding orientations and interaction affinities [34,35,36]. Our objective was to identify and select the most stable pose for each compound, considering both binding affinity and interaction types. The receptor of interest in our study is Serine/threonine-protein kinase 10 (STK10), which was obtained from the RCSB protein database to assess the ligand-protein interaction. The ligand and protein structures were both prepared. For the protein, the water molecules were removed, Kollman charges were added, Gasteiger charges were computed, and hydrogen atoms (only polar) were added. The prepared protein structure was saved as a PDBQT file. Regarding the ligands, the root was detected, torsions were selected, and the structures were saved as PDBQT files [37,38]. The Autodock system utilizes a three-dimensional grid that encompasses the active site of the 6I2Y protein, enabling rotational exploration of ligands within the site to expedite energy evaluation [39,40]. The coordinates of the grid box were set as X = 9.412 Å, Y = 20.328 Å, and Z = 24.168 Å, with dimensions of 72 * 78 * 92 Å^3^. We performed docking of the ligands with the Serine/threonine-protein kinase 10 (STK10) protein using Lamarckian Genetic Algorithms. We used the Protein Preparation Wizard (Protein Preparation Wizard) and Prime in Maestro to fill in the missing side chains for protein 6I2Y.

### 3.10. Molecular Dynamic

Molecular dynamics simulations have become a widely used method for investigating biomolecules, allowing for increased sampling time and more realistic boundary conditions. These simulations can now be performed on larger and more complex systems, including transmembrane channels. Simulations provide valuable insights into biochemical processes and add a dynamic perspective to structural data. In our study, a molecular dynamics (MD) simulation was conducted using Desmond software to validate the binding modes of ligands and gain a general understanding of protein-ligand complexes. An orthorhombic simulation box was prepared using the SPC water model, ensuring a minimum distance of 10 times between the protein’s surface and the box boundary [15,41,42]. To neutralize the charge of the solvated systems, Na^+^ and Cl^−^ counterions were added, adjusting the salt concentration to 0.15 M, which mimics physiological conditions. The system was gradually heated from zero to 300 K (at 1 bar pressure) using the Nose-Hoover thermal algorithm and the Martina–Tobias–Klein method. Isothermal-isobaric ensemble simulations were conducted for 100 ns. Analysis of the protein’s structure and dynamic behavior was performed using RMSD and RMSF plots.

### 3.11. Molecular Mechanics-Generalized Born Surface Area (MM-GBSA)

In this study, we employed the MM-GBSA (Molecular Mechanics-Generalized Born Surface Area) approach to recalibrate docking patterns derived from molecular docking simulations [16,43]. This procedure was conducted to evaluate the free binding energies of ligands binding to the active pocket of Serine/threonine-protein kinase 10 (STK10) protein. The objective was to gauge the binding efficacy of promising drug candidates with the target receptor (PDB code: 6I2Y) and anticipate the most favorable interactions that lead to the lowest free binding energy (${\Delta G}_{\mathrm{bind}}{=E}_{\mathrm{complexe}}{(\mathrm{minimized})+E}_{\mathrm{ligand}}{(\mathrm{minimized})-E}_{\mathrm{receptor}}\left(\mathrm{minimized}\right)$). The Prime MM-GBSA simulation involved minimizing the energies of complexes utilizing the OPLS3e force field and VSGB solvate model at pH 7 ± 2, facilitated through the MM-GBSA Prime package in Schrodinger 2020-3 73 [13]. During this setup, both the protein-ligand complexes and the ligands themselves underwent energy minimization. Consequently, the free binding energy (ΔG_bind_) of the analyzed systems could be determined.

## 4. Conclusions

In this study, we utilized 3D-QSAR to investigate the structure-activity relationship of quinoline derivative analogues as potential anticancer agents. The developed models demonstrated excellent predictive ability for a test set of ligands, indicating their reliability in predicting pIC_50_ values. By analyzing the CoMFA contour maps, we gained valuable insights into the structure-activity relationship and identified key structural features influencing the ligands’ activity. The robust performance of the external validation further validated the reliability of our models, providing confidence in the predicted activities of the newly designed ligands. Building upon the structure-activity relationships derived from this study, we designed new derivatives. To evaluate their potential, we employed the POM method to calculate molecular properties, predict bioactivity, and assess toxicity. The results of the POM method indicated that all designed ligands exhibited activity within the range of standard drugs, demonstrating their promise as potential candidates. Subsequently, molecular docking was employed to explore the interactions between the designed ligands and the protein (PDB: 6I2Y). The results revealed favorable binding and indicated good stability of the designed ligands within the active sites of the protein.

To further investigate their dynamic behavior, we performed MD simulations on the best docking configurations, specifically focusing on ligand 7, for a duration of 100 ns. These simulations confirmed the stability of the ligands within the protein’s active sites over the simulation time. In general, this comprehensive approach combining 3D-QSAR, molecular design, POM analysis, molecular docking, MD simulations, and MM-GBSA free Energy calculations provides valuable insights into the structure-activity relationship and stability of the designed ligands within the protein’s active sites. These findings support the potential of the newly designed ligands as promising anticancer agents.

## Figures and Tables

**Table 1:** PLS statistical parameters for CoMFA model.

PLS Model	Statistical Parameters					Fractions	
	R^2^	Q^2^	SEE	N	R_ext_^2^	Ster	Elec
CoMFA	0.913	0.625	0.073	3	0.875	0.515	0.485

**R^2^:** Non-cross-validated correlation coefficient; **Q^2^:** Cross-validated correlation coefficient. **R_ext_^2^:** External validation correlation coefficient; **SEE:** Standard error of the estimate; **N:** Optimal number of components; **F:** F-test value.

**Table 2:** Experimental in vitro and predicted in silico biological inhibitory activity pIC_50_ data for the investigated ligands.

Ligand	pIC_50_ Observed	pIC_50_ Predicted	Residuals	Ligand	pIC_50_ Observed	pIC_50_ Predicted	Residuals
	In Vitro	In Silico			In Vitro	In Silico	
**3a**	5.1	5.2	−0.1	**21a**	5.69	5.73	−0.04
**3b**	5.61	5.7	−0.09	21b *	6.64	6.76	−0.12
**4a**	5.58	5.64	−0.06	**21c**	6.38	6.49	−0.11
**4b**	5.06	5.2	−0.14	**21d**	6.3	6.46	−0.16
**6a**	5.36	5.46	−0,1	22a *	5.9	5.98	−0.08
**6b**	6.6	6.49	−0.14	**22b**	5.9	5.98	−0.08
**7a**	5.22	5.38	−0.16	**22c**	6.72	6.69	0.03
7b *	6.49	6.57	−0.08	22d *	6.46	6.57	−0.11
**10**	5.2	5.46	−0.26	**24a**	6.02	6.17	−0.15
**12a**	5.17	5.13	0.04	**24b**	6.08	6.19	−0.11
12b *	6.55	6.52	0.03	**24c**	6.1	6.34	−0.24
**14**	6.59	6.67	−0.08	**24d**	6.14	6.11	0.03
**16**	6.49	6.59	−0.1	24e *	6.21	6.39	−0.18
**18a**	6.04	6.18	−0.14	**24f**	6.42	6.53	−0.11
18b *	6.31	6.42	−0.11	**24g**	6.21	6.18	0.03
20a *	6.02	6.15	−0.13	**24h**	6.49	6.4	0.09
**20b**	6.28	6.23	0.05	**-**	-	-	-

* Test set compounds.

**Table 3:** The results of Y-randomization test.

Iteration	CoMFA	
	Q^2^	r^2^
1	−0.37	0.84
2	0.23	0.68
3	0.10	0.72
4	−0.3	0.84
5	−0.03	0.59
6	−0.22	0.82
7	−0.27	0.83
8	0.16	0.72
9	0.25	0.71
10	−0.33	0.65
11	0.291	0.55
12	−0.05	0.58
13	−0.15	0.54
14	0.08	0.61
15	−0.12	0.63

**Table 4:** Ligands designed and their predicted biological inhibitory activity using 3D-QSAR/CoMFA model.

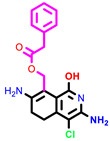		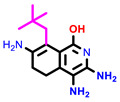		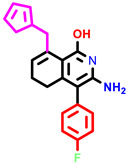	
1	pIC_50_ = 6.98	2	pIC_50_ = 7.01	3	pIC_50_ = 6.87
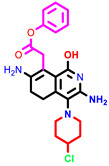		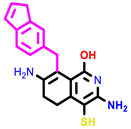		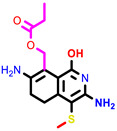	
4	pIC_50_ = 6.64	5	pIC_50_ = 7.1	6	pIC_50_ = 7.04
		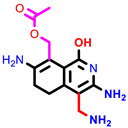			
		7	pIC_50_ = 7.23		

**Table 5:** Osiris calculations of the designed ligands.

Ligands	MW	Toxicity Risks				Osiris Calculations			
		MUT	TUMO	IRR	REP	c-LogP	Logs	DL	DS
**1**	345.0	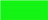	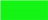	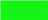	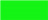	2.58	−4.7	−2.62	0.38
**2**	262.0	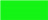	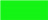	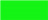	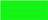	1.53	−3.57	−5.06	0.44
**3**	289.0	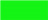	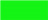	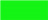	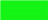	2.19	−4.18	−2.61	0.42
**4**	352.0	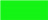	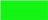	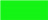	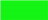	1.09	−3.68	2.4	0.49
**5**	337.0	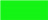	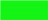	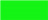	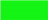	2.55	−5.81	1.23	0.3
**6**	323	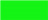	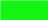	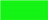	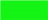	1.46	−3.9	4.46	0.33
**7**	278	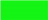	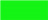	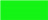	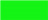	0.93	−2.48	1.6	0.54
Non-toxic			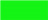						

MUT: Mutagen; TUMO: Tumorigenic; IRR: Irritant; REP: Effect on reproduction; DL: Druglikness; DS: Drug-Score.

**Table 6:** Molinspiration calculations of the designed ligands.

Ligands	Molinspiration Calculations				Drug-Likeness					
	TPSA	NONH	NV	VOL	GPCRL	ICM	KI	NRL	PI	EI
**1**	118.99	3	1	459.83	−0.42	−0.71	−0.65	−0.71	−0.48	−0.14
**2**	111.19	5	1	254.94	−0.22	0.3	0.02	−0.96	−0.31	−0.42
**3**	59.14	3	0	303.1	0.53	0.5	0.53	−0.08	0.09	0.63
**4**	114.71	7	0	376.35	−0.03	−0.08	−0.00	−0.74	−0.12	−0.2
**5**	85.17	5	0	299.91	0.5	0.14	0.27	0.00	0.1	0.67
**6**	111.47	5	0	273.48	−0.06	−0.05	−0.2	−0.33	−0.12	−0.41
**7**	137.49	7	1	250.08	0.04	0.01	0.11	−0.94	0.03	0.45

TPSA: Total Polar Surface Area NONH: Number of OH--N or O--NH interactions. NV: No. of Lipinski’s five rules’ violations; VOL: Volume; GPCRL: GPCR ligand; ICM: Ion Channel Modulator; KI: Kinase Inhibitor; NRL: Nuclear Receptor Ligand; PI: Protease; Protease Inhibitor; EI: Enzyme Inhibitor.

**Table 7:** Protein-ligand interactions of designed ligands 1–7 and template 22c towards STK10’s active pocket.

Ligands	Binding Affinity (Kcal/mol)	Interaction Hydrogen- Binding	Hydrophobic Interaction
**22c**	−4.3	Carbon-Hydrogen bond: Val A294 Arg A139 Conventional H-bond: Glu A290	π-sigma: Leu A289 alkyl and π-alkyl: Ala A286
**1**	−6.8	Conventional H-bond: Cys B206, Asp B215, Met B205, Tyr B214	alkyl and π-alkyl: Ala B218, Met A205 π-sulfur: Cys B206.
**2**	−5.1	Conventional H-bond: Gln A140, Ile A297;	amide stackers: Ser299; Vander wals: Asn A300.
**3**	−7.5	-	alkyl and π-alkyl: Lys B159, Ala B117 π-π stacked: Phe B176 Halogen: Asp B119
**4**	−6.3	Conventional H-bond: Arg A139, Tyr A92, Gln A140.	alkyl and π-alkyl: Pro A91.
**5**	−5.6	Conventional H-bond: Glu B280, Asn B278	alkyl and π-alkyl: Pro B279, Ala218
**6**	−5.2	Conventional H-bond: Asn A300, Tyr A92	π-anion: Asp A170
**7**	−7.9	Conventional H-bond: Asn A146, Ser A150, Ala A285, Ser A284, Gln A287.	alkyl and π-alkyl: Ala A286.

**Table 8:** MM-GBSA-free binding energy for STK10-ligand7 and 22c systems.

Complex	ΔG_bind_ (MM-GBSA)	ΔG_bind_ Hbond	ΔG_bind_ VdW
STK10-7	−20.9293	−2.9044	−0.3727
STK10-22c	−13.6075	−1.1743	0.48926

**Table 9:** Structure-activity dataset of targeted Quinoline derivatives.

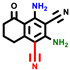		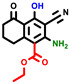		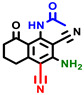		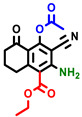		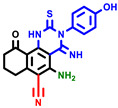		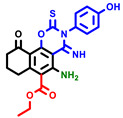		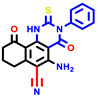	
**3a**	pIC_50_ = 5.1	3b	pIC_50_ = 5.61	4a	pIC_50_ = 5.58	4b	pIC_50_ = 5.06	6a	pIC_50_ = 5.36	6b *	pIC_50_ = 6.35	7a	pIC_50_ = 5.22
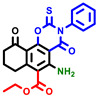		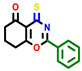		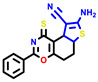		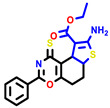		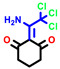		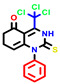		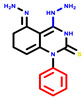	
7b *	pIC_50_ = 6.49	10	pIC_50_ = 5.2	12a	pIC_50_ = 5.17	12b *	pIC_50_ = 6.55	14	pIC_50_ = 6.59	16	pIC_50_ = 6.49	18a	pIC_50_ = 6.04
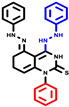		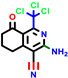		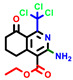		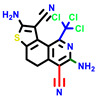		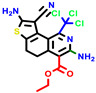		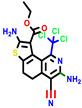		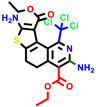	
18b *	pIC_50_ = 6.31	20a *	pIC_50_ = 6.02	20b	pIC_50_ = 6.02	21a	pIC_50_ = 5.69	21b *	pIC_50_ = 6.64	21c	pIC_50_ = 6.38	21d	pIC_50_ = 6.3
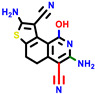		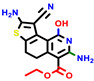		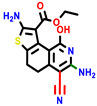		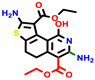		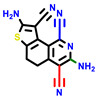		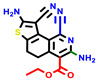		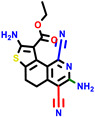	
22a *	pIC_50_ = 5.9	22b	pIC_50_ = 5.9	22c	pIC_50_ = 6.72	22d *	pIC_50_ = 6.46	24a	pIC_50_ = 6.02	24b	pIC_50_ = 6.08	24c	pIC_50_ = 6.10
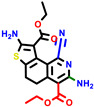		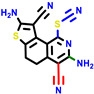		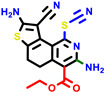		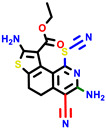		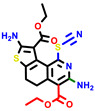					
24d	pIC_50_ = 6.14	24e *	pIC_50_ = 6.21	24f	pIC_50_ = 6.24	24g	pIC_50_ = 6.21	24h	pIC_50_ = 6.49				

* Test set compounds.

## Data Availability

Data are contained within the article.
